# Genetic dissection of maize plant architecture with an ultra-high density bin map based on recombinant inbred lines

**DOI:** 10.1186/s12864-016-2555-z

**Published:** 2016-03-03

**Authors:** Zhiqiang Zhou, Chaoshu Zhang, Yu Zhou, Zhuanfang Hao, Zhenhua Wang, Xing Zeng, Hong Di, Mingshun Li, Degui Zhang, Hongjun Yong, Shihuang Zhang, Jianfeng Weng, Xinhai Li

**Affiliations:** Institute of Crop Science, Chinese Academy of Agricultural Sciences, Zhongguancun South Street, Haidian District, Beijing, 100081 China; College of Agronomy, Northeast Agricultural University, Mucai Street, XiangFang District, Harbin, Heilongjiang 150030 China

**Keywords:** Maize, Ultra-high density bin map, Plant architecture, Genotyping by sequencing, Quantitative trait loci

## Abstract

**Background:**

Plant architecture attributes, such as plant height, ear height, and internode number, have played an important role in the historical increases in grain yield, lodging resistance, and biomass in maize (*Zea mays* L*.*). Analyzing the genetic basis of variation in plant architecture using high density QTL mapping will be of benefit for the breeding of maize for many traits. However, the low density of molecular markers in existing genetic maps has limited the efficiency and accuracy of QTL mapping. Genotyping by sequencing (GBS) is an improved strategy for addressing a complex genome via next-generation sequencing technology. GBS has been a powerful tool for SNP discovery and high-density genetic map construction. The creation of ultra-high density genetic maps using large populations of advanced recombinant inbred lines (RILs) is an efficient way to identify QTL for complex agronomic traits.

**Results:**

A set of 314 RILs derived from inbreds Ye478 and Qi319 were generated and subjected to GBS. A total of 137,699,000 reads with an average of 357,376 reads per individual RIL were generated, which is equivalent to approximately 0.07-fold coverage of the maize B73 RefGen_V3 genome for each individual RIL. A high-density genetic map was constructed using 4183 bin markers (100-Kb intervals with no recombination events). The total genetic distance covered by the linkage map was 1545.65 cM and the average distance between adjacent markers was 0.37 cM with a physical distance of about 0.51 Mb. Our results demonstrated a relatively high degree of collinearity between the genetic map and the B73 reference genome. The quality and accuracy of the bin map for QTL detection was verified by the mapping of a known gene, *pericarp color 1* (*P1*), which controls the color of the cob, with a high LOD value of 80.78 on chromosome 1. Using this high-density bin map, 35 QTL affecting plant architecture, including 14 for plant height, 14 for ear height, and seven for internode number were detected across three environments. Interestingly, pQTL10, which influences all three of these traits, was stably detected in three environments on chromosome 10 within an interval of 14.6 Mb. Two MYB transcription factor genes, GRMZM2G325907 and GRMZM2G108892, which might regulate plant cell wall metabolism are the candidate genes for *qPH10*.

**Conclusions:**

Here, an ultra-high density accurate linkage map for a set of maize RILs was constructed using a GBS strategy. This map will facilitate identification of genes and exploration of QTL for plant architecture in maize. It will also be helpful for further research into the mechanisms that control plant architecture while also providing a basis for marker-assisted selection.

**Electronic supplementary material:**

The online version of this article (doi:10.1186/s12864-016-2555-z) contains supplementary material, which is available to authorized users.

## Background

Plant architecture directly affects biomass in higher plants, and particularly influences grain yields in agricultural crops. The genetics of various aspects of maize (*Zea mays* L.) plant architecture, a complicated agronomic trait that is mainly determined by plant height (PH), ear height (EH), and internode number (IN), have recently been extensively investigated [1–3]. These three components reflect the spatial conformation of the maize plant, which is closely correlated with biomass, lodging resistance, and tolerance of stress associated with high plant density. Therefore, improved plant conformation not only increases maize productivity and thereby yield, but also assists breeding efforts to coordinate the sometimes contradictory manifestation of traits in populations compared to an individual plants [4, 5]. For specific maize varieties, plant height under certain circumstances may be associated with increased internode number and reduced ear height, thus achieving a lower center of gravity and enhancing lodging resistance [6]. So in developing cultivars for maize breeding, it is crucial to optimize these three components of plant architecture while avoiding yield losses.

The first genetic linkage map of maize was constructed in 1986 based on restriction fragment length polymorphisms (RFLP) in the F_2_ mapping population of a cross between H427 and 761 [7]. Subsequently, the advent of PCR-based markers, such as simple sequence repeats (SSRs) [8], expressed sequence tags (ESTs) [9], and amplified fragment length polymorphisms (AFLPs) [10] supplied greater impetus to detect DNA polymorphism and generate a highly saturated genetic linkage map in maize. Using these genetic maps, numerous quantitative trait loci (QTL) for many complex agronomic traits have been identified and mapped to all 10 maize chromosomes [11, 12]. However, most of the genetic maps based on low-throughput molecular markers are of low density, which limits the efficiency and accuracy of QTL mapping and reduces the coverage of genetic markers for maize breeding [13]. The resolution of QTL mapping largely depends on marker density, and population size and types [14, 15]. Compared with other populations such as early generation populations, the development, genotyping, and phenotyping of advanced-generation RIL populations for QTL mapping of crop species is very costly and time-consuming. Because RILs are permanent populations, all of the homozygous lines of which they are comprised can be tested at various points over multiple years, which can help refine understanding of the genetics of a trait and increase the accuracy of QTL detection in many environments. In comparison to the other kinds of segregating populations, RILs have particular advantages, such the absence of dominance effects, fewer genetic parameters, and lower experimental error. RILs are therefore an ideal type of population for quantitative trait analysis.

Single-nucleotide polymorphisms (SNPs) are the most abundant form of genetic variation in genomes [16, 17]. Next-generation sequencing technologies have made it possible to develop numerous SNP markers for genotyping large populations, which has successfully accelerated the genetic analysis of various crop species, such as maize, rice, barley, wheat, and soybean [18–20]. Recently, a set of 1359 maize developed using Sequenom technology were genotyped in recombinant inbred lines from the IBM population to generate a SNPs-based genetic map [21]. Genotyping by sequencing (GBS) is a powerful strategy for assessing large, complex genomes that has proven a useful tool for SNP discovery and genetic mapping [22]. With the same sequence data, the accuracy of a marker increases as the sequencing depth for a single locus increases. GBS not only cuts the cost of sequence-based genetic analyses through the use of techniques that initially reduce genome complexity, but can markedly reduce SNP imputation errors via a modified sliding-window approach [18], by which adjacent SNPs with the same genotype in an interval are combined into bins (100-Kb intervals with no recombination events) that demarcate recombination events across the entire population. Bins can then be used as markers to construct an ultra-high density genetic map. Recently, this strategy was used with an F_2_ and the US-NAM population to increase the marker density of consensus maize genetic maps that were constructed with 6533 and 5296 markers, respectively [23, 24]. Both of these maps were shown to be more powerful for detecting QTL than were traditional methods and they have also been used to fine map tassel and ear architecture loci and identify the genetic determinants of flowering-time.

Plant architecture and its three components are among the most heritable traits that can be genetically manipulated. Several approaches have been successfully used for the genetic analysis of plant architecture, such as map-based cloning and association mapping. To date, using different linkage mapping populations, more than 245 QTL for traits related to plant architecture have been identified on different chromosomal regions of the maize genome, such as bins 1.07, 3.01, 3.03–3.04, and 5.05–5.06 (2010 December update to Gramene QTL database). For instance, a maize introgression library that was produced using Gaspe’ Flint as the donor and B73 as the receptor, had a much lower number of internodes than did B73. Using this library, four QTL for IN were detected as major QTL related to plant architecture, indicating that variation in internode number drives variation in plant architecture [25, 26]. In addition, genome-wide association analysis (GWAS), by which genetic and phenotypic variation in a population is evaluated to discover new marker-phenotype associations, has also been used to scan for novel loci affecting maize plant architecture [27].

Maize plant architecture, especially plant height, is a complicated quantitative trait controlled by a large number of genes that encode proteins involved in hormone synthesis, transport, and signaling, such as *an1*, *br2*, *d1*, *d2*, *d3*, *d5*, *d8*, *d9*, *qPH3.1*, *DWF1*, and *DWF4* [28–34]. Most of these genes play roles in the metabolism of the plant hormone gibberellic acid (GA) and are involved in diverse aspects of plant growth and development, especially internode elongation [35, 36]. In addition, the constituents of plant primary and secondary walls such as cellulose, hemicelluloses, and pectin strongly influence overall plant architecture, growth, and development [37]. For example, maize *cellulose synthase-like D1* (*ZmCSLD1*) is essential for plant cell division, and affects cell number and size by acting during the early phases of cross-wall formation. Thus, mutants in this gene have characteristic phenotypes including narrow and warty leaves and stems [38]. Recently, the MYB transcription factor family was identified as a member of the transcriptional network regulating secondary wall biosynthesis in xylem tissues of *Arabidopsis*, and was proposed to act on cellulose and lignin biosynthesis [39–41]. However, due to the adverse effects on grain yield and various reproductive abnormalities associated with these genes or mutants, they are not particularly useful in maize breeding to control plant architecture.

In the present study, therefore, the objectives were (1) to identify bin markers from high-throughput GBS data in a set of 314 recombinant inbred lines (RILs) derived from two maize elite inbred lines Ye478 and Qi319 in China; (2) to construct a high-density linkage map based on these bin markers and to validate the map using well-characterized genes from the reference genome; (3) to map QTL for plant height, ear height, and internode number combining their phenotypes in the RILs across three environments, and to validate the detected QTL using maize gene annotations.

## Results

### Resequencing parental lines and GBS of RILs

**Fig. 1:**
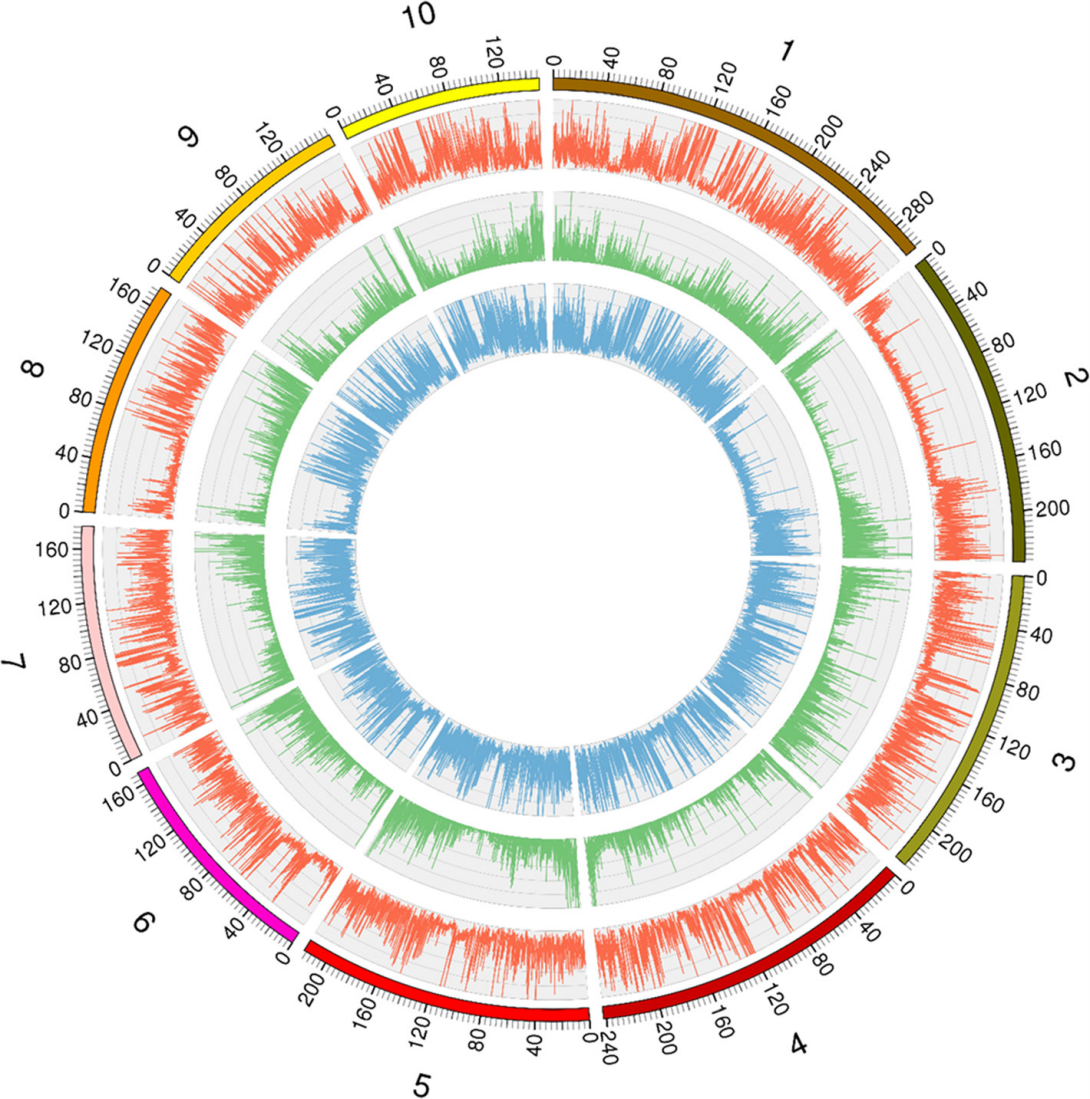
Genome-wide distribution of SNPs and genetic variants throughout the Ye478 and Qi319 genomes. The outermost box with scale represents the 10 maize chromosomes. The orange histogram represents the density of SNPs that are polymorphic between Ye478 and Qi319; the green histogram represents the density of polymorphic SNPs within coding sequences between Ye478 and Qi319; the blue histogram indicates the density of insertions or deletions (Indels) between Ye478 and Qi319

Maize elite inbred lines Ye478 and Qi319, the two founder lines of the RILs used in the present study, were sequenced at effective sequencing depths of about 29.5-fold and 33.7-fold, respectively. For Ye478 and Qi319, 678,819,425 and 803,698,828 reads were mapped to the B73 RefGen_V3 genome, and a total of 5,400,526 and 6,909,395 SNPs were identified, respectively. Further analysis revealed 3,549,088 homozygous polymorphic SNPs and 429,116 homozygous polymorphic Indels between Ye478 and Qi319 (Fig. 1).

**Fig. 2:**
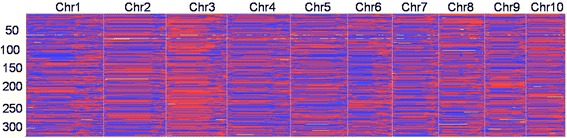
Recombination bin map of the RIL population derived from Ye478 and Qi319. The bin map is comprised of 4183 bin markers inferred from 88,268 high-quality SNPs mapped in the RIL population. Physical position is based on B73 RefGen_V3 sequence. Red: Qi319 genotype; blue: Ye478 genotype; yellow: heterozygote

The RIL population was then genotyped using GBS technology. The enzyme digestion was highly efficient and 90.9 % complete. A total of 137,699,000 reads were generated. On average, there were 357,376 reads per individual, which is equivalent to approximately 0.07-fold coverage of the maize genome. The overall GC content of the sequences was about 41.8 % and Q20 scores were about 92.1 %. Because the two parents are homozygous inbred lines with genotypes of *aa* and *bb*, only 3,549,088 homozygous polymorphic SNPs fell into the *aa* × *bb* segregation pattern. Based on the reference parental polymorphic loci, a total of 164,919 SNPs were identified by low-coverage sequencing of the RIL population. In a population comprised of RILs, SNPs should segregate in a 1:1 ratio. After filtering out SNPs exhibiting significant segregation distortion (*p* < 0.001, *X*^2^ test), a total of 88,268 SNPs were retained to determine bin markers (Additional file 1: Figure S1).

### Genetic linkage map with bin markers

The breakpoints in the RIL population were determined using a sliding-window approach [18] in which genotypes were called based on SNP ratios. A total of 12,835 breakpoints were identified for 314 RILs with 40.87 breakpoints per individual, which corresponds to the number of breakpoints identified using GBS data in the US-NAM and CN-NAM populations by Li et al. [24]. (Additional file 1: Figure S2). To conduct genetic analysis, the recombination maps were divided into a skeleton bin map and all chromosomes of the 314 RILs were aligned and compared over minimum 100-Kb intervals (Figure 2). Adjacent 100-Kb intervals with the same genotype across the entire RIL population are considered a single recombination bin. Thus a total of 4183 recombination bins were determined, which indicated that the vast majority of recombination events could be captured in the RIL population.

The physical lengths of the recombination bins ranged from 100 Kb to 21 Mb, with an average of 492 Kb and a median of 200 Kb (Table 1). A total of 79.7 % of these bins were less than 0.5 Mb in length and 8.4 % of bins ranged from 1 Mb to 10 Mb in length (Additional file 1: Figure S3). Seventeen bins were greater than 10 Mb in length, 15 of which were located in centromeric or pericentromeric regions with suppressed recombination (Additional file 1: Table S1). The other two long recombination bins, mk853 and mk855, were located in regions of very low SNP coverage but high recombination rates on chromosome 2, in the physical positions 141.9–153.4 Mb and 153.9–166.8 Mb, respectively (Additional file 1: Figure S1).

**Table 1:** Characteristics of the high-density genetic map derived from a cross between Ye478 and Qi319

Chr.^a^	No. markers^b^	Physical distance (Mb)	Genetic distance (cM)	Avg. distance between markers (cM)	<5 cM Gap	Max. gap (cM)
1	738	301.43	239.48	0.32	738	4.81
2	337	237.89	151.46	0.45	336	4.93
3	476	232.23	163.33	0.34	474	5.57
4	447	242.03	163.22	0.37	445	6.51
5	487	217.93	170.86	0.35	486	2.52
6	346	169.38	120.38	0.35	344	5.11
7	395	176.81	143.24	0.36	394	2.94
8	358	175.35	142.66	0.4	357	2.96
9	323	157.02	122.05	0.38	321	11.15
10	276	149.63	128.97	0.47	274	5.11
Total	4183	2059.7	1545.65	0.37	4169	11.15

^a^Chr., indicates chromosome

^b^No.markers, the number of markers on chromosome

A high-density genetic map was constructed by mapping these 4183 bin markers onto the 10 maize chromosomes (Additional file 1: Figure S4). About 0.4 % of genotypic data was missing. The total genetic distance of the linkage map was 1545.65 cM. The average distance between two adjacent markers was 0.37 cM, which corresponds to a physical distance of about 0.51 Mb. For chromosome 1, there were 738 bin markers covering a genetic length of 239.48 cM, which was the longest genetic length covered among the 10 maize chromosomes. In contrast, for chromosome 6 there were 346 bin markers that covered 120.38 cM, the shortest genetic length covered in this map. There were five gaps that ranged from 5 cM to 12 cM in length and the largest gap of 11.15 cM was on chromosome 9 (Table 1).

### The quality and accuracy of the map

**Fig. 3:**
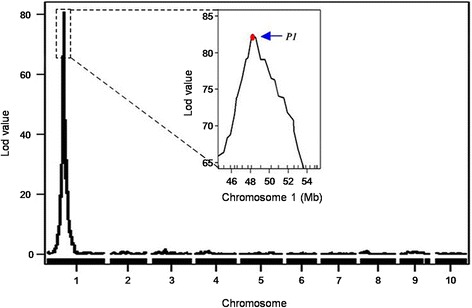
Mapping of *P1*, which controls cob color, in the RIL population. Curves in plot indicate the genetic coordinates along chromosomes or the physical coordinates within a chromosome (*x-axis*) and LOD score (*y-axis*) of the detected QTL. Mapping curve of the QTL that controls cob color of is located on chromosome 1; the box shows a magnification of the peak on chromosome 1. The red dot represents the relative physical position of the *P1* gene

To assess the quality and accuracy of this genetic map, bin markers were mapped to the maize B73 RefGen_V3 reference genome. The scatter plot of markers in the 10 linkage groups aligned well with the B73 reference chromosomes, which indicated excellent collinearity between the maize B73 reference genome and these bin markers (Additional file 1: Figure S5). In order to evaluate the power and accuracy of this genetic map for a highly heritable trait, QTL analysis of cob color was performed in the RIL population. The QTL *qC1*, whose peak encompassed the cloned gene *pericarp color 1* (*P1*) [42] was detected on chromosome 1 with a high LOD value of 80.78 (Fig. 3). *P1* regulates red pigmentation in cob, pericarp, tassel glumes, and husks, and is located at mk187 on chromosome 1 at position 48.1 Mb.

### Phenotypic features of plant height, ear height, and internode number

**Fig. 4:**
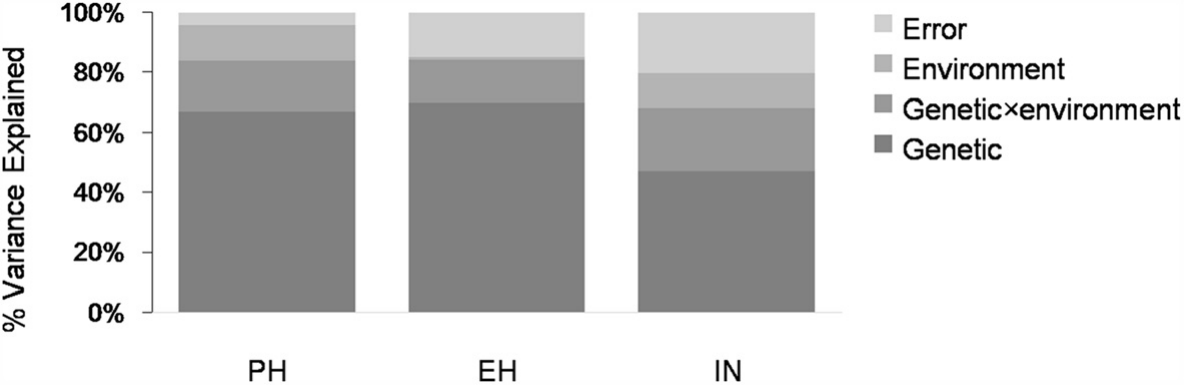
Variation in PH, EH, and IN was attributed to genetic and environmental factors across the RIL population. The different shades of grey in the stacked bar diagram indicate the various factors that explain phenotypic variance. PH: plant height; EH: ear height; IN: internode number

**Fig. 5:**
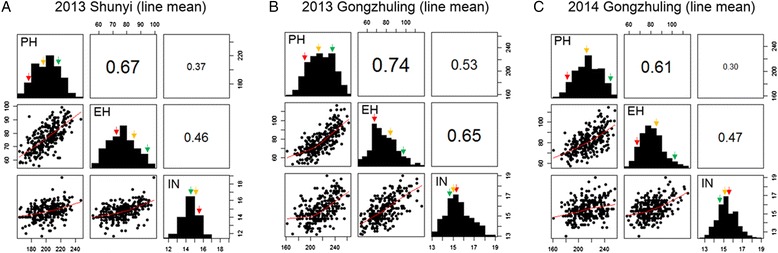
Correlations between variation in PH, EH, and IN. Positive correlations between PH and EH were greater among line means than those with IN across all three environments. PH: plant height; EH: ear height; IN: internode number. Red arrow: Ye478; green arrow: Qi319; orange arrow: mid-parent. **a**, 2013 Shunyi; **b**, 2013 Gongzhuling; and **c**, 2014 Gongzhuling

Tremendous phenotypic variation was observed for PH, EH, and IN in the RIL population across the three environments (Table 2). Analysis of variance for plant architecture was performed to detect the sources of phenotypic variation. Phenotypic variances were significantly influenced by both genetic and environmental factors (Fig. 4). However, the fraction of variation attributable to genotype-by-environment interaction was still significantly greater for IN than for the other two traits. Estimates of broad-sense heritability indicated that a proportion of genetic variance was attributable to the phenotypic variance of the entire RIL population. These estimates were high for all traits across three environments. The most heritable trait across the RIL population was PH, for which the heritability was 94.87 % (Table 2), followed by EH (85.24 %) and IN (82.33 %). The largest positive correlation of values between lines occurred between PH and EH (*r* = 0.68, *P* < 2.2e-16), which showed a uniform trend across three environments (Fig. 5). Due to the environmental effect, the QTL for plant architecture were identified separately in each environment. The loci detected in common across multiple environments were considered as consistent QTL for plant architecture.

**Table 2:** Phenotypes of the parental lines and RIL population across three environments

Trait^a^	Env.^b^	Ye478	Qi319	RIL population					
				Range	Mean ± SD^c^	Skewness	Kurtosis	CV^d^ (%)	Heritability (%)
PH	E1	177.80	218.00	163.50–247.40	200.92 ± 16.38	0.01	−0.63	8.15	93.20
	E2	187.00	237.00	175.17–266.00	217.58 ± 20	0.03	−0.74	9.19	99.06
	E3	180.47	248.53	161.00–255.50	208.71 ± 17.48	−0.03	−0.10	8.38	92.35
EH	E1	72.80	96.50	61.63–100.10	80.05 ± 7.81	0.14	−0.48	8.15	83.30
	E2	68.20	98.08	50.20–116.13	77.65 ± 13.13	0.49	−0.03	9.19	86.37
	E3	68.50	101.00	51.18–116.50	77.81 ± 13.06	0.49	0.01	8.38	86.06
IN	E1	15.47	14.64	11.67–18.80	14.66 ± 0.92	0.08	1.48	10.10	84.18
	E2	15.20	14.73	13.17–19.00	15.61 ± 1.19	0.36	−0.34	12.90	80.61
	E3	15.67	14.67	12.50–19.00	15.54 ± 1.02	0.19	0.25	12.03	82.19

^a^Trait is the name of the component of plant architecture: PH, plant height; EH, ear height; IN, internode number

^b^Env., the specific environment: E1 is 2013 Shunyi; E2 is 2013 Gongzhuling; and E3 is 2014 Gongzhuling

^c^SD, standard deviation

^d^CV, coefficient of variation

### Bin markers associated with plant height, ear height, and internode number

**Fig. 6:**
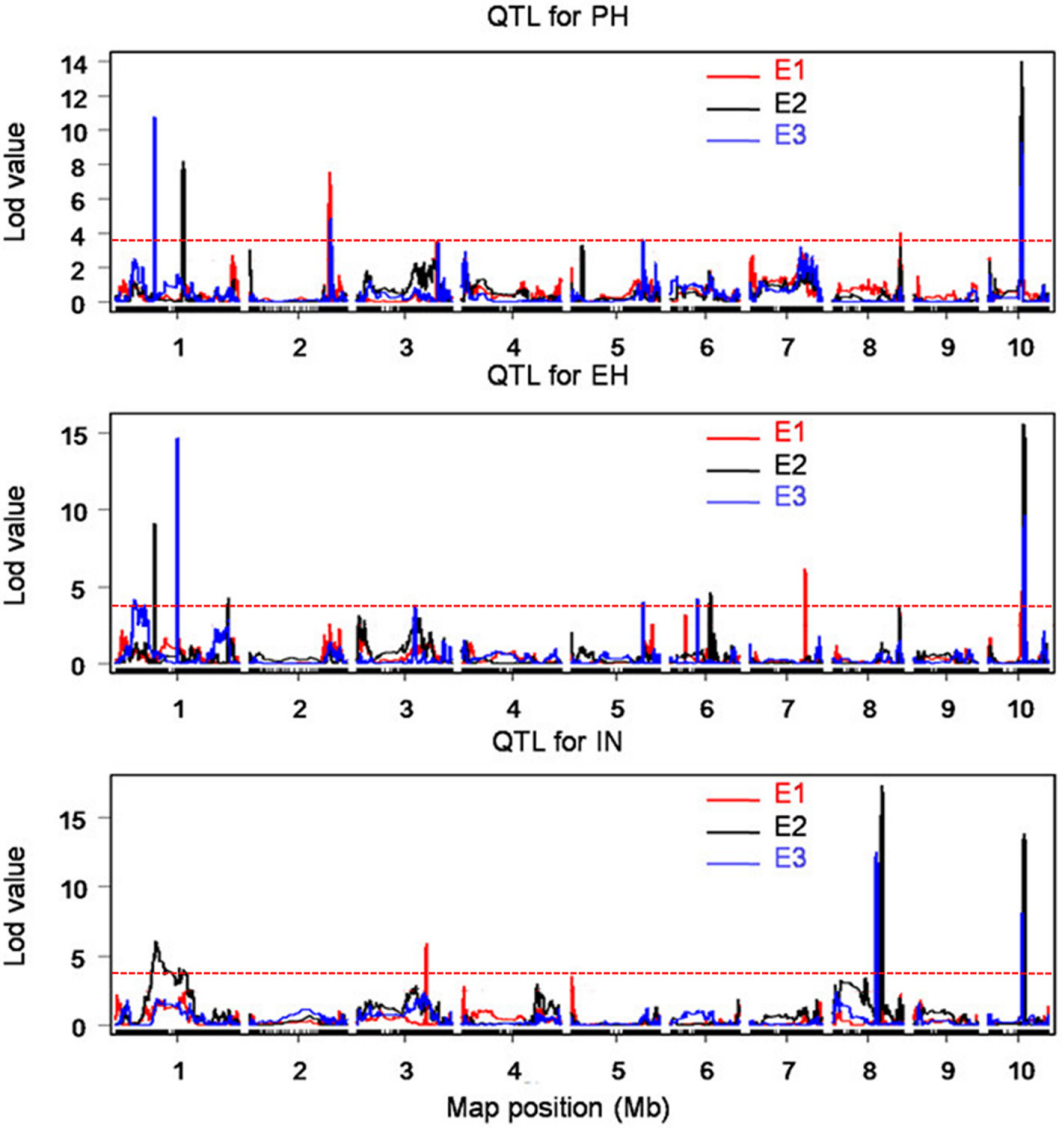
Mapping of QTL on ten chromosomes for PH, EH, and IN across three environments. The curves indicate the physical position (*x-axis*) of bin markers against LOD score (*y-axis*) of QTL detected on ten chromosomes. Different colors represent different environments: E1, 2013 Shunyi; E2, 2013 Gongzhuling; and E3, 2014 Gongzhuling. The red dashed lines present the LOD threshold. PH: plant height; EH: ear height; IN: internode number

QTL for the three plant architecture components in each environment were detected in the bin map using the *R/qtl* package implemented in R software [43]. A total of 35 QTL were identified: 14 of them influence PH and are distributed on chromosomes 1, 2, 4, 5, and 10; 14 of them influence EH and are distributed on chromosomes 1, 3, 5, 6, 7, 8, and 10; and 7 of them influence IN and are distributed on chromosomes 1, 3, 8, and 10 (Fig. 6). The confidence intervals for these 35 QTL spanned physical distances from 3.8 Mb to 46.9 Mb, with an average of 10.65 Mb by comparison to the B73 RefGen_v3 genome. The phenotypic variation explained by each QTL ranged from 2.60 to 15.68 % of the variation in a trait, with means of 6.17, 6.89, and 12.50 % for PH, EH, and IN, respectively (Table 3). In addition, when QTL for the three plant architecture components were detected by analysis in three environments combined, only a total of 10 QTL were identified. Three of them influence PH, three of them influence EH, and four of them influence IN (Additional file 1: Figure S8). The confidence intervals for these 10 QTL spanned physical distances from 2.65 Mb to 7.5 Mb, with an average of 5.01 Mb by comparison to the B73 RefGen_v3 genome. The phenotypic variation explained by each QTL ranged from 3.88 to 20.13 % of the variation in a trait, with means of 9.23, 10.15, and 10.24 % for PH, EH, and IN, respectively (Additional file 1: Table S2). These QTL include some of the QTL that also can be detected in a single environment.

**Table 3:** QTL identified for PH, EH, and IN using high-density bin map

Trait Name^a^	Name^b^	Effect^c^	Chr.^d^	Flanking marker^e^	Interval^f^ (Mb)	Physical length^g^ (Mb)	LOD^h^	PVE^i^	ADD^j^
PH	*qPH1-1*	E1,E2	1	mk328–mk336	161.10–169.60	8.5	6.42	7.44	10.49
	*qPH1-2*	E3	1	mk271–mk280	91.00–98.40	6.3	10.63	12.01	13.98
	*qPH2*	E1,E2,E3	2	mk926–mk950	193.10–201.40	8.3	5.31	5.91	9.22
	*qPH4*	E3	4	mk1569–mk1610	3.20–16.20	13.35	8.95	2.6	6.51
	*qPH5-1*	E2	5	mk2112–mk2119	22.70–29.10	5.75	7.4	4.13	8.64
	*qPH5-2*	E3	5	mk2312–mk2433	175.20–207.50	32.05	3.53	4.23	8.32
	*qPH8*	E1,E2	8	mk3521–mk3550	166.00–170.20	4.2	3.58	3.81	−7.09
	*qPH10*	E1,E2,E3	10	mk4016–mk4026	81.30–85.10	3.8	9.09	9.35	11.93
EH	*qEH1-1*	E1,E2	1	mk271–mk280	91.00–98.40	6.3	8.72	9.96	7.16
	*qEH1-2*	E3	1	mk309–mk314	146.50–154.90	6.85	14.63	15.68	8.75
	*qEH3*	E2,E3	3	mk1278–mk1284	138.70–145.30	5.9	3.68	3.43	−4.58
	*qEH5*	E3	5	mk2302–mk2320	172.90–178.60	5.45	3.98	3.9	4.28
	*qEH6-1*	E2	6	mk2598–mk2610	95.30–101.10	5.45	4.62	4.78	5.75
	*qEH6-2*	E3	6	mk2541–mk2549	60.90–72.20	7.4	4.23	4.16	4.59
	*qEH7*	E1	7	mk3037–mk3044	133.00–137.60	4.25	6.15	6.26	4.73
	*qEH8-1*	E2	8	mk3401–mk3531	120.30–167.40	46.9	3.68	3.92	−5.17
	*qEH10*	E1,E2,E3	10	mk4012–mk4037	80.10–94.70	14.6	9.99	9.9	7.14
IN	*qIN1*	E2	1	mk271–mk295	91.00–111.70	19.4	6.26	6.48	0.61
	*qIN3*	E1	3	mk1339–mk1357	167.60–173.00	5.25	6.54	8.11	−0.52
	*qIN8-1*	E1,E2	8	mk3405–mk3412	121.90–127.40	5.5	13.08	15.53	−0.83
	*qIN8-2*	E3	8	mk3371–mk3382	106.30–112.50	5.9	12.5	18.73	−0.91
	*qIN10*	E2,E3	10	mk4016–mk4036	81.30–94.20	12.9	11.11	13.66	0.84

^a^Trait is the name of the component of plant architecture: *PH* plant height, *EH* ear height, *IN* internode number

^b^The name of each QTL is a composite of the influenced trait: PH, EH, IN

^c^The effect of each QTL in a specific environment: E1 is 2013 Shunyi; E2 is 2013 Gongzhuling; and E3 2014 is Gongzhuling

^d^Chr., chromosome

^e^Flanking markers, the markers to the left and right of the QTL

^f^Interval, confidence interval between two bin markers

^g^Physical length, interval between the two markers on the B73 genome

^h^LOD, the logarithm of odds score

^i^PVE, the phenotypic variance explained by individual QTL

^j^ADD, the additive effect value. The LOD scores, PVE values, and ADD values are shown as mean values for QTL with multiple effects

Nine QTL regions were consistently detected in more than two environments, and thus were viewed as stable QTL in this study. Four of the nine stable QTL influence PH, three of them influence EH, and two of them influence IN. *qPH10* had the largest effect of the five stable QTL influencing PH and explained 9.35 % of the phenotypic variation in PH. Alleles from Qi319 increased PH by 11.93 cm. The genetic length of the *qPH10* region was about 2.26 cM, which corresponds to a physical distance of about 3.8 Mb in B73 RefGen_v3 (Additional file 1: Figure S6). *qEH1-1*, which explained 9.96 % of the phenotypic variation in EH, had the largest effect of the three stable QTL for that trait. Alleles from Qi319 increased the ear height by 14.28 cm. The genetic length of the *qEH1-1* region was about 1.76 cM, corresponding to a physical distance of about 6.30 Mb. Only two stable QTL for IN were identified, with *qIN8-2* having the largest effect and explaining a total of 15.53 % of the phenotypic variation. Ultimately, a total of six pleiotropic QTL (pQTL) distributed on chromosomes 1, 3, 5, 8, and 10 were obtained by integrating the 35 QTL for the three traits (Table 4). Notably, pQTL10, which was detected for all three traits across more than two environments, was located within the region between mk4012 and mk4037, corresponding to a physical distance of about 14.6 Mb in the present study (Additional file 1: Figure S7).

**Table 4:** Pleiotropic QTL (pQTL) for three plant architecture traits in three environments

pQTL^a^	Chr.^b^	Flanking marker^c^	Interval^d^ (Mb)	Physical length^e^ (Mb)	NO. of QTLs	Integrated QTLs
pQTL1	1	mk271–mk295	91.00–111.70	20.7	4	qPH1-2, qEH1-1, qIN1
pQTL3	3	mk1278–mk1431	138.70–201.00	62.3	3	qEH3-2, qIN3-2
pQTL5	5	mk2302–mk2320	172.90–178.60	5.7	2	qPH5-2, qEH5
pQTL8-1	8	mk3401–mk3531	120.30–167.40	47.1	3	qEH8-2, qIN8-1
pQTL8-2	8	mk3510–mk3550	164.70–170.20	5.5	3	qPH8, qEH8-1
pQTL10	10	mk4012–mk4037	80.10–94.70	14.6	8	qPH10, qEH10, qIN10

^a^The pleiotropic QTL name

^b^Chr., chromosome

^c^Flanking markers, the markers to the left and right of the QTL

^d^Interval, confidence interval between two bin markers

^e^Physical length, interval between the two markers on the B73 genome

### Candidate gene prediction

According to the maize gene annotation database accessible at MaizeGDB (http://www.maizegdb.org), the shortest physical intervals of *qPH10* encompassed 45 protein-coding genes, but only seven protein functions had so far been annotated in this region (Additional file 1: Table S3). Among the candidate genes within the 1.5-LOD drop on either side of the peak bin that delimits the *qPH10* interval, are MYB transcription factors GRMZM2G325907 and GRMZM2G108892 that might regulate plant height.

## Discussion

QTL mapping has been an efficient strategy for the dissection of quantitative trait in maize breeding [23]. However, the quality of genetic maps significantly affects the accuracy of the QTL mapping. Increasing the density of markers distributed around the entire genome improves the resolution of genetic maps [44]. Nevertheless, the linkage disequilibrium (LD) of maize is about 300 Kb, which is significantly lower than that of other plants. It is critical to increase marker density and improve the resolution of genetic maps for QTL mapping [27, 45]. With the development of next-generation sequencing technology and the complete re-sequencing of the whole genome of the maize elite inbred line B73, numerous SNP markers have become useful for high-density genetic map construction in maize [46, 47].

GBS is a cost-effective, rapid, informative, and reliable genotyping method for assessing large, complex genomes for SNP discovery and genotyping [48]. The advantage of this technology is that prior genome information is not required for inference or map construction [23], although imputation of SNPs can become more accurate in bi-parental mapping populations when a reference genome is available for tested species [18]. In the present study, the average effective depth of sequencing of the populations derived from these two parents was approximately 31.6-fold and the re-sequencing error rate was only 0.05 %. This result suggests that the genome sequences of the parents were of high quality, and that the accuracy of genotype calling was relatively insensitive to even the relatively high error rates of low-coverage sequence data [18]. Because of the quality of the parental sequence information, the inferior SNP calls in their progenies could be found and filtered out. We also genotyped RILs in their F_8_ to F_10_ generations using 114 SSR markers and constructed a linkage map. This linkage map had average marker coverage of about 13.1 cM, corresponding to a physical distance of about 17.5 Mb. In contrast to PCR-based SSR markers, a recombination breakpoint between two bin markers that are an average of about 0.51 Mb apart can be identified by GBS, which represents a 34-fold improvement in the resolution of recombination breakpoints. Additionally, a higher average coverage of one marker per 0.37 cM achievable by GBS would reduce the chance of missing any double-crossovers in the mapping population that were not identified by a PCR-based method [18].

Based on the GBS results we obtained with Illumina short-read sequencing technology, we constructed a genetic map of a maize RIL population derived from Qi319 and Ye478 using 4183 bin markers representing 88,268 SNP markers. Within the RIL population, we observed a region on chromosome 2 of approximately 24 Mb that had less informative markers (Additional file 1: Figure S1). The lack of informative markers in this large region likely indicates a region of identity-by-descent (IBD) that was not efficiently disrupted by recombination during artificial selection [49]. Recently, Chen et al. [23] and Wen et al. [50] developed a high-density integrated genetic linkage map for maize by compositing the SNP data obtained from F_2_ and RIL mapping populations, respectively. The two maps consisted of 6533 and 2496 markers and spanned a genomic map distance of 1396 cM and 1790.2 cM, respectively. The mean genetic distances between adjacent bin markers in their maps were 0.2 cM and 0.72 cM, respectively. Compared to those RIL population-based genetic maps, our genetic map covered a similar distance in terms of genome size but had more markers, thus the mean genetic distance between adjacent markers was narrowed to 0.37 cM. This means that a GBS-based SNP genetic map can detect more recombination events in larger RIL populations, which would increase the total number of bins while reducing bin size. In addition, compared with previous studies, our population allowed detection of more breakpoints than did RIL-based genetic maps in rice, wheat, barley and, soybean [19, 20, 51]. Moreover, construction of our high-density genetic map was based on a single RIL population, thus QTL mapping could be performed more conveniently and efficiently than in the F_2_ population for a given phenotypic trait. This is because F_2_ populations require much more frequent calling between homozygous and heterozygous genotypes, and maps based on F_2_ populations have higher error rates than do maps based on RILs. With high-density genetic maps and the high-quality sequence of the B73 genome, we can more accurately predict candidate genes within narrow regions between two adjacent bin markers when high genomic collinearity between the genetic map and the maize reference genome is identified for a region. Our results demonstrated a relatively high collinearity between our genetic map and the B73 reference genome (Additional file 1: Figure S5). This indicates that identifying candidate genes through comparative mapping will be feasible. Finally, the quality and accuracy of the bin map for QTL detection was verified by the mapping of *P1*, which regulates red pigmentation in cob, pericarp, tassel glumes, and husks.

Plant architecture is the final expression of the spatial distribution of plant organs in maize. It is important to attain reasonable spatial distribution of plant organs to improve the photosynthetic performance and yield of maize grown in high-density fields [52]. Previous studies have detected QTL for plant architecture traits, particularly plant height and ear height, on the 10 chromosomes of maize [2, 26, 53]. Most of these results showed that only a few QTL have consistent and strong additive effects in different environments or populations. However, most QTL for plant architecture are affected by environmental factors and are under the control of several genes with minor effects. However, only stable and highly heritable QTL are useful for MAS, which might take place in different environments and even different genetic backgrounds.

In the present study, a RIL population comprised of 314 families was used to map QTL for plant height, ear height, and internode number in three environments. The results showed that the genetics of these components of plant architecture are complex. A total of 35 QTL were detected in the present study, including three QTL that could be detected in all three environments, six QTL that could be detected in two environments and 10 QTL that could be detected by combined analysis. These stable and consistent QTL could be considered priority candidates for MAS. The QTL identified here, including *qPH1-1*, *qPH2*, *qPH8*, and *qPH10* for PH; *qEH1-1*, *qEH3*, and *qEH10* for EH; and *qIN8-1* and *qIN10* for IN, are likely important QTL for these plant architecture-related traits (Fig. 6). Meanwhile, there are six pQTL regions, such as pQTL10 that might show pleiotropy or tight linkage to other QTL. The regions in which these QTL are located might also represent hot spots for important QTL that control plant height and ear height closely linked to QTL influencing other traits, such as loci for that control flowering time. According to the maize gene annotation database at MaizeGDB, the physical interval containing pQTL10 encompassed at least five genes that affect flowering traits, such as *FIE*, *CHI* and *ZmCCT* [54], which have been cloned and functionally verified, and homologs of known flowering-related genes from other species. Understanding the functions of the genes in these co-localizing regions will help breeder achieve the full yield potential of maize. Notably, the present study also detected a number of separate QTL controlling either plant height or ear height, but not both. For example, *qPH2* only controls plant height, *qEH3* only controls ear height, and *qIN8-1* only controls internode number. Plant height, ear height, and internode number each still seem to have a relatively independent genetic basis, and are likely subject to different mechanisms of genetic regulation. Therefore, the fine mapping of these QTL and validation of the potential candidate genes may be a reliable and feasible strategy for QTL cloning to isolate loci that may be even more valuable for maize breeding. Our results provide important information for such further fine mapping to find quantitative trait genes and may help reveal the molecular mechanisms responsible for plant architecture.

The QTL-by-environment interaction (G × E, QEI) may be due to the specific expression of certain genes during the process of adaptation to different ecological environments. Li et al. [55] suggested that three conditions could occur: (1) the specific QTL might be expressed in one environment, but not in another environment; (2) the specific QTL might be strongly expressed in a certain environment and weakly expressed in another environment; (3) or the specific QTL could have opposite effects in different environments. In our study, ten QTL for PH, EH, and IN were insensitive to different environments and these QTL were detected in multiple environments, which indicated that the QTL-by-environment interaction had a smaller effect. This could be for at least two reasons. The first reason is that QTL that explain a higher of phenotypic variation also have larger direct effects on phenotypes and smaller genotype-by-environment interaction effects. The second reason is that QTL that explain lower proportions of phenotypic variation tend to have larger genotype-by-environment interaction effects and can be overlooked using current statistical methods [56]. Previous studies demonstrated that QTL × environment interaction are an important property of many QTL, even for highly heritable traits such as plant height and that these interactions are trait- and gene-specific [55]. When detected in multiple environments, the main effects of most QTL are consistent in direction but vary considerably in magnitude across multiple environments. In addition, it remains unclear whether inconsistent QTL detection is due to type-II error arising from the use of single minimum thresholds or to true differential trait expression across environments. So information about QTL × environment interaction should be considered particularly carefully when performing marker-assisted manipulation of plant architecture, especially for more environment-specific QTL. [57].

In comparison to the other kinds of segregating populations, such as early generation populations, RILs have particular advantages, such the absence of dominance effects, fewer genetic parameters, and lower experimental error. With the rapid development of GBS technology, the reduction of QTL intervals in dense marker maps for fine mapping is valuable for better defining candidate genes underlying mapped loci. Among the QTL we detected, the minimum physical interval for *qPH10* was 3.80 Mb, which suggests that this approach is highly efficient for the identification and mapping of QTL relative to traditional methods. This analysis of the hot spots for maize plant architecture will provide an important reference for the cloning of key genes involved in this set of traits. For example, according to the maize gene annotation data at MaizeGDB (http://www.maizegdb.org), the shortest physical intervals of *qPH10* encompassed 45 protein-coding genes, but only seven protein functions had so far been annotated in this region (Additional file 1: Table S3). Recent studies in *Arabidopsis* and rice identified the MYB transcription factor family as a member of the transcriptional network regulating secondary wall biosynthesis in xylem tissues and as an actor in cell wall formation and plant growth [39]. Among the candidate genes within the 1.5-LOD drop on either side of the peak bin that delimits the *qPH10* interval, are MYB transcription factors GRMZM2G325907 and GRMZM2G108892 that might regulate plant height. These results will not only promote further research into the mechanisms that control plant height, ear height, and internode number of maize, but will also provide a basis for MAS of these traits, the development of elite inbred lines, and the breeding of hybrids. Further, we propose that QTL mapping using GBS in large RIL populations is a highly efficient way to rapidly identify useful alleles present in germplasm. Elucidating the genetic control of complex traits could substantially accelerate crop improvement in a cost-effective fashion.

## Conclusions

In the present study, we constructed an ultra-high density maize linkage map after large-scale development of markers by GBS of an RIL population. These results showed that this high-density map is accurate enough to use for efficient QTL mapping. Using this map, we mapped three plant architecture traits in maize and identified major QTL in three environments. Further, within these QTL, two candidate genes could be predicted. Future studies leading to additional phenotype annotations for introgressed genomic regions would accelerate the identification and accumulation of known QTL and genes related to the development of plant architecture in maize.

## Methods

### Plant materials and phenotyping

A RIL mapping population consisting of 314 F_11_ individuals was derived from the selfed cross of maize elite inbred lines Qi319 as male and Ye478 as female. Each plot contained one row 4 m long and 0.6 m wide, with a total of 17 plants at a density of 60,000 plants/ha. Two replicates were conducted with a randomized incomplete block design. For QTL analysis, phenotypic data for PH, EH, and IN were determined as the mean of measurements from five randomly selected individuals per RIL. The two parents and their progeny were grown at three locations (location/years) in China, including Gongzhuling Experimental Station (N43°52′, E124°82′) in 2013 and 2014, and Shunyi Experimental Station (N40°13′, E116°34′) in 2013. These location/year combinations were designated as E1, E2, and E3, respectively. In all environments, PH was measured as the distance in centimeters from the soil to the top of the plant at reproductive maturity; EH was scored as the distance from the soil to the primary ear node, at the same developmental stage. IN was scored as the number of internodes between the top brace root internode and the top of the plant, including the part of tassel, but excluding any variation in brace root internodes and any subterranean internodes [58].

Broad-sense heritability (*H*^*2*^) of plant height-related traits across multiple environments was calculated according to Knapp et al. [59]. Heritability was calculated as: *H*^*2*^ = *δ*^*2*^_*g*_/(*δ*^*2*^_*g*_ + *δ*^*2*^_*ge*_/*e* + *δ*^*2*^/*e* × *r*), where *δ*^*2*^_*g*_ is the genetic variance, *δ*^*2*^_*ge*_ is genotype × environment interaction, *δ*^*2*^ is the error variance, *e* is the number of environments, and *r* is the number of replications per environment. The estimates for *δ*^*2*^_*g*_, *δ*^*2*^_*ge*_, and *δ*^*2*^ were obtained by standard analysis of variance (ANOVA) using the general linear model procedure (PROC GLM) in Statistical Analysis System (SAS) software 9.0. Estimates of the variance components associated with all terms in the model were calculated using PROC MIXED. Spearman rank correlation coefficients and related statistics were calculated using *R* software.

### DNA extraction

The core panel of 314 RILs for genotyping was planted in May 2014 at the Chinese Academy of Agricultural Sciences, Beijing, China. Young, healthy leaves from two parents and each of 314 RIL individuals were collected and frozen in liquid nitrogen, ground in a SPEX GENO 2010 GRINDER®, and then transferred to a −80 °C freezer. Total genomic DNA was extracted from each parental and RIL leaf sample following the manufacturer’s protocols with the Plant Genomic DNA Kit (TIANGEN, Beijing, China). The integrity and quality of the extracted DNA was evaluated by electrophoresis on 1 % agarose gels run with a λ DNA ladder size standard ladder, and the nucleic acid concentration of each sample was determined using a Qubit® 2.0 Fluorometer (Invitrogen, Carlsbad, USA) and NanoDrop 2000 (Thermo Scientific, MA, USA).

### Genotyping by high-throughput sequencing

For each of the two parents, a total of 1.5 μg DNA was used as input material for the DNA sample preparations. Sequencing libraries were generated as described by Cheng et al. [60]. These parental libraries were sequenced on an Illumina HiSeq 2000 platform and 125-bp paired-end reads with insert sizes of around 350 bp were generated.

The genomic DNAs from the each of the RIL lines were incubated with *MseI* (New England Biolabs, Ipswitch, MA), T4 DNA ligase (NEB), ATP, and the Y-adapter N containing a barcode. The digestion was conducted at 37 °C and heated at 65 °C to inactivate the enzymes. Restriction digestion-ligation reactions were completed in the same tube, and then further digested with *NlaIII* (NEB) and *EcoRI* (NEB) at 37 °C. The restriction digestion-ligation samples were purified using the Agencourt AMPure XP System. Each clean read was checked using a Perl script to identify whether a read begins with a TAA site that can be recognized by the restriction enzyme *MseI*. The percent completeness of enzyme digestion equals the number of clean reads that contain a TAA site divided by the total number of clean reads times 100. The efficiency of enzymatic digestion for each sample was calculated in this manner. PCR amplifications were carried out in a single tube with purified samples and Phusion Master Mix (NEB) after adding universal primer and index primer to each sample. The PCR reactions were purified using Agencourt AMPure XP (Beckman) and pooled, then run out on a 2 % agarose gel. Fragments of 350 to 400 bp (including indexes and adaptors) in size were isolated using a Gel Extraction Kit (Qiagen, Valencia, CA). These fragments were then purified using the Agencourt AMPure XP System, then diluted for sequencing. Finally, paired-end sequencing was performed on the selected tags using an Illumina 2500 platform (Illumina, USA) by Novogene Bioinformatics Institute, Beijing, China.

### Sequence data grouping and SNP identification

The sequences of each individual RIL were sorted according to the barcoded adapters. Four stringent filtering steps were carried out: 1) removing reads with ≥ 10 % unidentified nucleotides (N); 2) removing reads with > 50 % bases having phred quality scores of < 5; 3) removing reads with > 10 nt aligned to the barcode adapter, allowing ≤ 10 % mismatches; and 4) removing reads that contain *NlaIII* or *EcoRI* cut site remnant sequences.

To identify SNPs in the RIL population, the Burrows-Wheeler Aligner (BWA) was used to align the clean reads from each sample against the reference genome with the settings ‘mem -t 4 -k 32 -M -R’, where -t is the number of threads, −k is the minimum seed length, −M is an option used to mark shorter split alignment hits as secondary alignments, and -R is the read group header line [61]. Alignment files were converted into BAM files using the sort setting in SAMtools software [62]. Variant calling was performed for all samples using the SAMtools software. SNPs were filtered using a custom Perl script and those exhibiting segregation distortion or sequencing errors were discarded. In order to determine the physical positions of each SNP, the software tool ANNOVAR [63], was used to align and annotate SNPs or InDels based on the GFF3 files from the B73RefGen_V3 sequence (ftp://ftp.ensemblgenomes.org/pub/release-20/plants/fasta/zea_mays/dna/Zea_mays.AGPv3.20.dna.toplevel.fa.gz). Polymorphic parental markers were classified into eight segregation patterns, such as ab × cd, ef × eg, hk × hk, lm × ll, nn × np, aa × bb, ab × cc and cc × ab, but aa and bb would be considered for the RIL population. However, variants with a heterozygous SNP call and unexpected base due to sequencing errors would be considered missing data.

### Bin map construction

Chi-square (*Χ*^2^) tests were conducted for all SNPs to detect segregation distortion. A sliding-window approach was applied for variant calling errors and to calculate the ratio of SNP alleles derived from Ye478 and Qi319 [18]. Genotypic data was scanned with a window size of 15 SNPs and a step size of one SNP. Windows with 11 or more SNPs from either parent were considered to be homozygous but those with fewer SNPs from a single parent were considered heterozygous. Adjacent windows with the same genotypes were combined into a single block, whereas adjacent blocks with different genotypes were assumed to be at or near a recombination breakpoint. A bin marker was designated when consecutive 100-Kb intervals lacked a recombination event in the entire population. For construction of the linkage map, the genetic distance between bin markers was determined using a Kosambi mapping function and the *est.map* function in the *R/qtl* package [43]. A Perl SVG module was used to draw the linkage map.

### QTL analysis using high-density genetic map

QTL for plant architecture in three environments were detected by composition-interval mapping using the *R/qtl* package. The threshold of LOD scores for evaluating the statistical significance of QTL effects was determined using 1000 permutations and a threshold *p* value of 0.05 with the *mqmpermutation* function in *R/qtl*. With 1000 permutations, a LOD score of 3.5 was considered the minimum to declare the presence of a QTL in a particular genomic region. The confidence interval for each QTL was assigned as a 1.5-LOD drop relative to the peak LOD for each bin. The filtered working gene list of the maize genome was downloaded from MaizeGDB (http://www.maizegdb.org) to identify possible candidate genes within each QTL. We selected the most likely candidate within the confidence interval by testing for either associations with gene function or associations between the gene and the pathways in which the phenotype is involved. Other analyses of genotypic data and construction of figures were also performed using *R* software.

### Availability of supporting data

The B73RefGen_V3 sequence supporting the results of this article is available in the Ensembl Genomes repository (ftp://ftp.ensemblgenomes.org/pub/release-20/plants/fasta/zea_mays/dna/Zea_mays.AGPv3.20.dna.toplevel.fa.gz). The filtered working gene list of the maize genome was downloaded from MaizeGDB (http://www.maizegdb.org).

## Additional file

Additional file 1:
**Figure S1.** Distribution of 88,268 high-quality SNPs identified from low-coverage sequencing of 314 individual RILs. 2. **Figure S2.** Recombination map of R001. **Figure S3.** Distribution of bin length. **Figure S4.** Comparison of the physical map and genetic maps created derived from 4183 bin markers. **Figure S5.** Collinearity analysis of maize marker linkage groups (lg) with the maize reference genome B73 RefGen_V3. **Figure S6.** Mapping of QTL controlling PH across three environments. **Figure S7.** Mapping of QTL controlling PH, EH, and IN across three environments to chromosome 10. **Figure S8.** Mapping of QTL for PH, EH, and IN on the ten maize chromosomes in three environments combined. ** Table S1.** Detailed information regarding the 17 bins that were greater than 10 Mb in length. **Table S2.** QTL identified for PH, EH, and IN with combined analysis of three environments. **Table S3.** Genes located in the intervals of *qPH10*. (DOC 2190 kb)

## Abbreviations

QTL: quantitative trait locus
GBS: genotyping by sequencing
RIL: recombinant inbred line
LG: linkage group
MAS: marker-assisted selection
RFLP: restriction fragment length polymorphism
AFLP: amplified fragment length polymorphism
SSR: simple sequence repeat
EST: expressed sequence tag
SNP: single nucleotide polymorphism
GWAS: genome wide association analysis
LOD: logarithm of odds
LD: linkage disequilibrium
IBD: identity-by-descent
RRL: reduced representation library
ANOVA: analysis of variance
SAS: statistical analysis system
QC: quality control
BWA: burrows-wheeler aligner
