# Supplementary material for: Genetic dissection of maize plant architecture with an ultra-high density bin map based on recombinant inbred lines
Source: BMC Genomics. 2016 Mar 3;17:178. doi: 10.1186/s12864-016-2555-z (PMC4778306; doi:10.1186/s12864-016-2555-z)
Supplement: Additional file 1: — Figure S1. Distribution of 88,268 high-quality SNPs identified from low-coverage sequencing of 314 individual RILs. 2. Figure S2. Recombination map of R001. Figure S3. Distribution of bin length. Figure S4. Comparison of the physical map and genetic maps created derived from 4183 bin markers. Figure S5. Collinearity analysis of maize marker linkage groups (lg) with the maize reference genome B73 RefGen_V3. Figure S6. Mapping of QTL controlling PH across three environments. Figure S7. Mapping of QTL controlling PH, EH, and IN across three environments to chromosome 10. Figure S8. Mapping of QTL for PH, EH, and IN on the ten maize chromosomes in three environments combined. Table S1. Detailed information regarding the 17 bins that were greater than 10 Mb in length. Table S2. QTL identified for PH, EH, and IN with combined analysis of three environments. Table S3. Genes located in the intervals of qPH10. (DOC 2190 kb) [file 12864_2016_2555_MOESM1_ESM.doc]

**Additional file 1**

**SUPPORTING INFORMATION**

**Figure S1** **Distribution of 88,268 high-quality SNPs identified from low-coverage sequencing of 314 individual RILs.** The physical positions below each chromosome are based on the B73 RefGen_v3 sequence. The short blue lines indicate SNP densities. The red point on each chromosome indicates the centromere. The red box indicates the largest region of low SNP density on chromosome 2.

**Figure S2** **Recombination map of R001.** All positions are transformed to physical positions according to the B73 RefGen_V3 sequence. Red lines: homozygous Qi319 genotype; Blue lines: homozygous Ye478 genotype.

**Figure S3** **Distribution of bin length.**

**Figure S4** **Comparison of the physical map and genetic maps created derived from 4183 bin markers.** The order of the bin markers depends on the physical position of each marker. The genetic map positions are on the left side of each linkage group (LG), and the marker names are along the right side of each linkage group.

**Figure S5** **Collinearity analysis of maize marker linkage groups (lg) with the maize reference genome B73 RefGen_V3.** The y-axis shows the linear order and genetic distances of the maize marker linkage groups, while the x-axis represents the linear order of physical position in the maize reference genome. All 4183 bin markers are plotted as a scatter diagram. Different colors indicate different chromosomes.

**Figure S6** **Mapping of QTL controlling PH across three environments.** The curves indicate the physical position (x-axis) of bin markers against LOD scores (y-axis) of QTL detected on chromosomes 10. Different colors represent different environments: E1, 2013 Shunyi; E2, 2013 Gongzhuling; E3, 2014 Gongzhuling. The red dashed lines present the LOD threshold. PH: plant height.

**Figure S7** **Mapping of QTL controlling PH, EH, and IN across three environments to chromosome 10.** The curves indicate the physical position (x-axis) of bin markers against LOD score (y-axis) of QTL detected on chromosome 10. Different colors represent different traits. The red dashed lines present the LOD threshold. PH: plant height; EH: ear height; IN: internode number. E1, 2013 Shunyi; E2, 2013 Gongzhuling; E3, 2014 Gongzhuling.

**Figure S8 Mapping of QTL for PH, EH, and IN on the ten maize chromosomes in three environments combined.** The curves indicate the physical position (x-axis) of bin markers against the LOD scores (y-axis) of QTL detected on each of ten chromosomes. The red dashed lines represent the LOD threshold. PH: plant height; EH: ear height; IN: internode number.

**Table S1 Detailed information regarding the 17 bins that were greater than 10 Mb in length.**

**Table S2 QTL identified on the ten maize chromosomes for PH, EH, and IN by combined analysis in three environments.**

**Table S3 Genes located in the intervals of *qPH10*.**

**
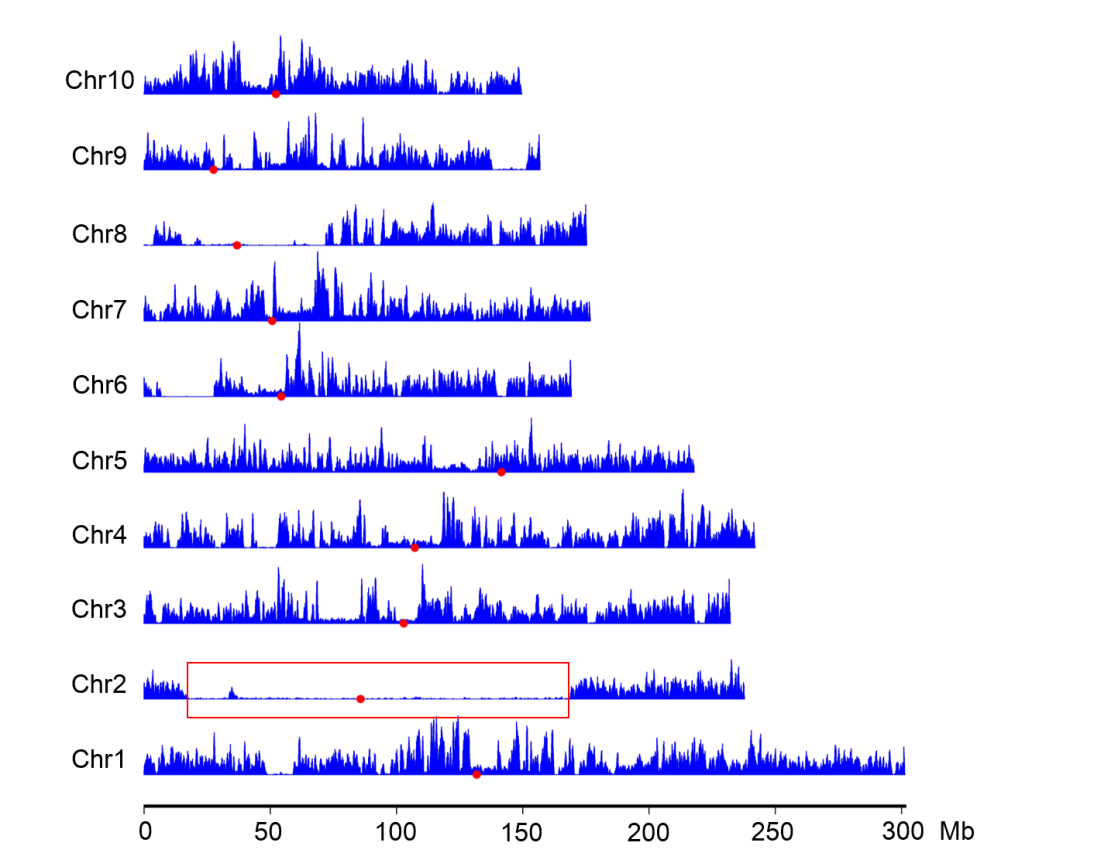
**

**Figure S1** **Distribution of 88,268 high-quality SNPs identified from low-coverage sequencing of 314 individual RILs.** The physical positions below each chromosome are based on the B73 RefGen_v3 sequence. The short blue lines indicate SNP densities. The red point on each chromosome indicates the centromere. The red box indicates the largest region of low SNP density on chromosome 2.


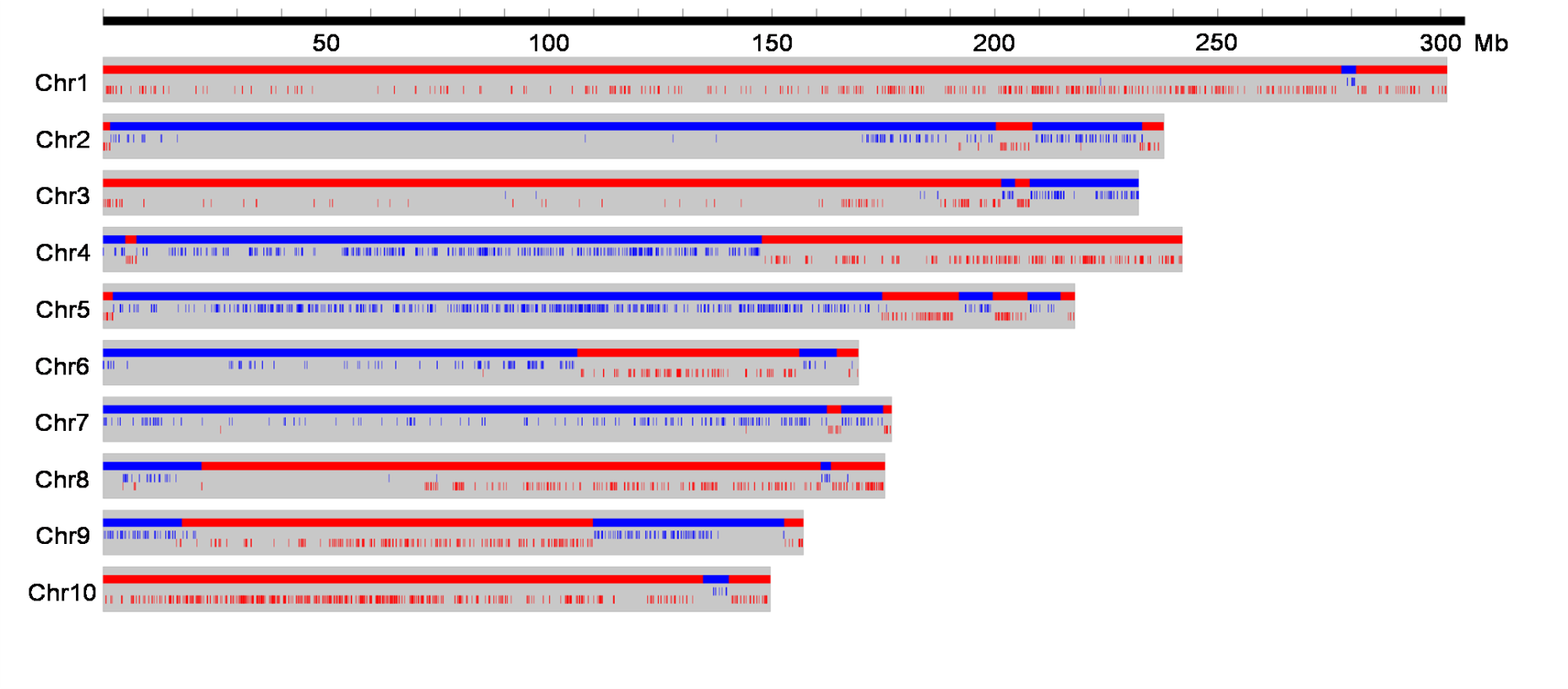


**Figure S2** **Recombination map of R001.** All positions are transformed to physical positions according to the B73 RefGen_V3 sequence. Red lines: homozygous Qi319 genotype; Blue lines: homozygous Ye478 genotyp


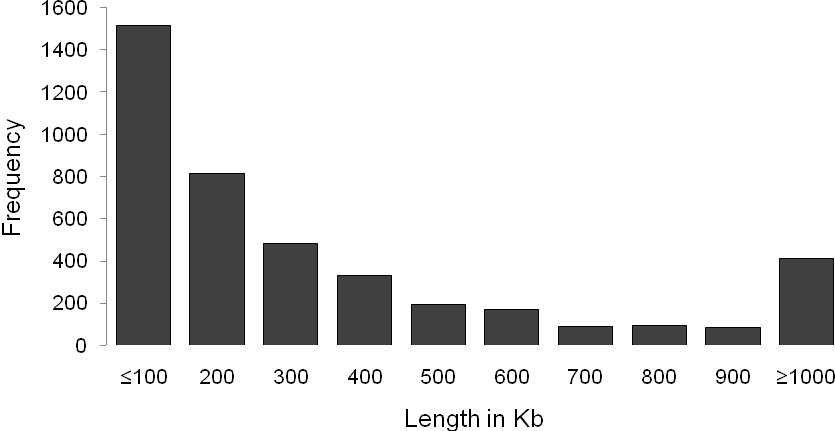


**Figure S3 Distribution of bin length.**

**
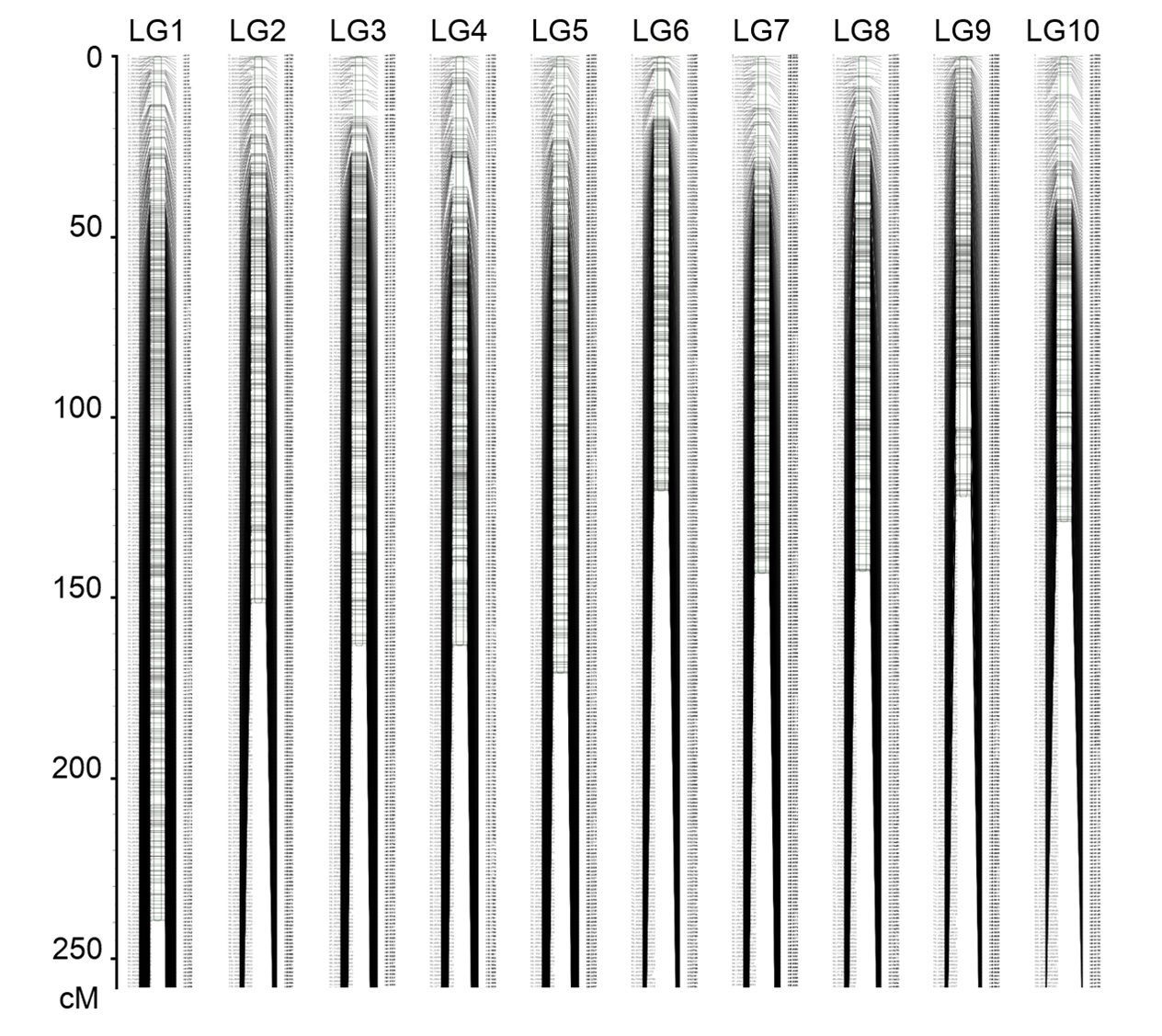
**

**Figure S4** **Comparison of the physical map and genetic maps created derived from 4183 bin markers.** The order of the bin markers depends on the physical position of each marker. The genetic map positions are on the left side of each linkage group (LG), and the marker names are along the right side of each linkage group.


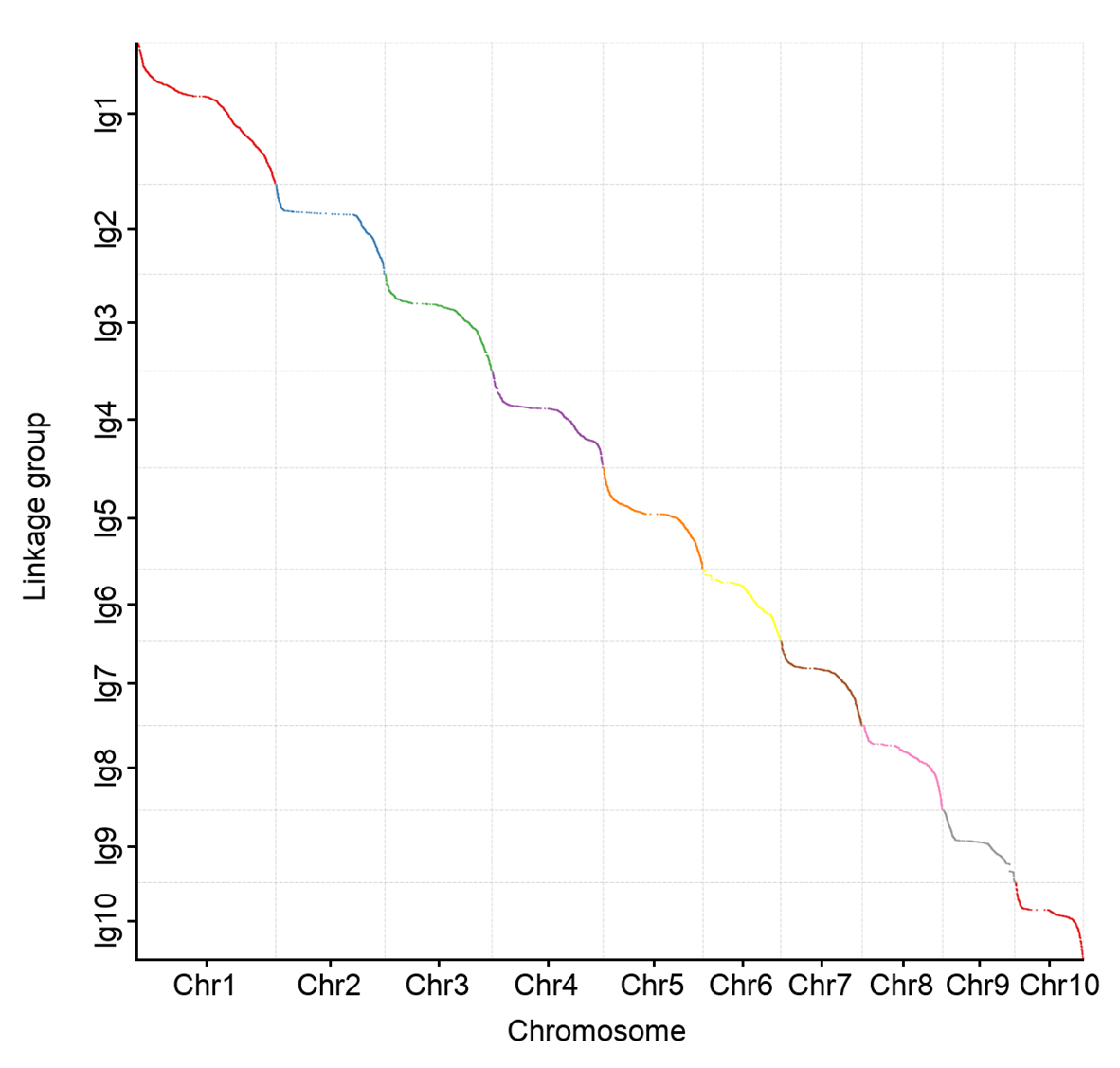


**Figure S5** **Collinearity analysis of maize marker linkage groups (lg) with the maize reference genome B73 RefGen_V3.** The y-axis shows the linear order and genetic distances of the maize marker linkage groups, while the x-axis represents the linear order of physical position in the maize reference genome. All 4183 bin markers are plotted as a scatter diagram. Different colors indicate different chromosomes.


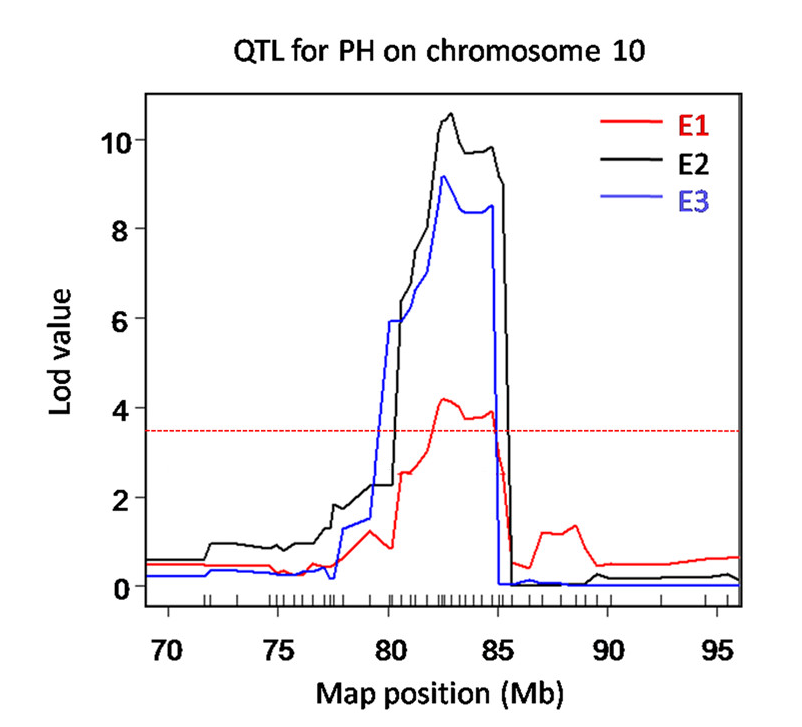


**Figure S6** **Mapping of QTL controlling PH across three environments.** The curves indicate the physical position (x-axis) of bin markers against LOD scores (y-axis) of QTL detected on chromosomes 10. Different colors represent different environments: E1, 2013 Shunyi; E2, 2013 Gongzhuling; E3, 2014 Gongzhuling. The red dashed lines present the LOD threshold. PH: plant height.


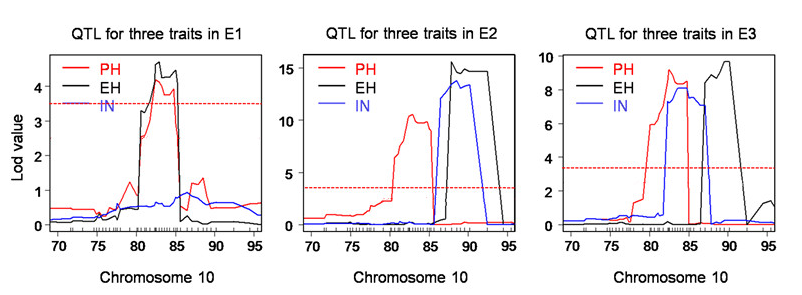


**Figure S7** **Mapping of QTL controlling PH, EH, and IN across three environments to chromosome 10.** The curves indicate the physical position (x-axis) of bin markers against LOD score (y-axis) of QTL detected on chromosome 10. Different colors represent different traits. The red dashed lines present the LOD threshold. PH: plant height; EH: ear height; IN: internode number. E1, 2013 Shunyi; E2, 2013 Gongzhuling; E3, 2014 Gongzhuling.


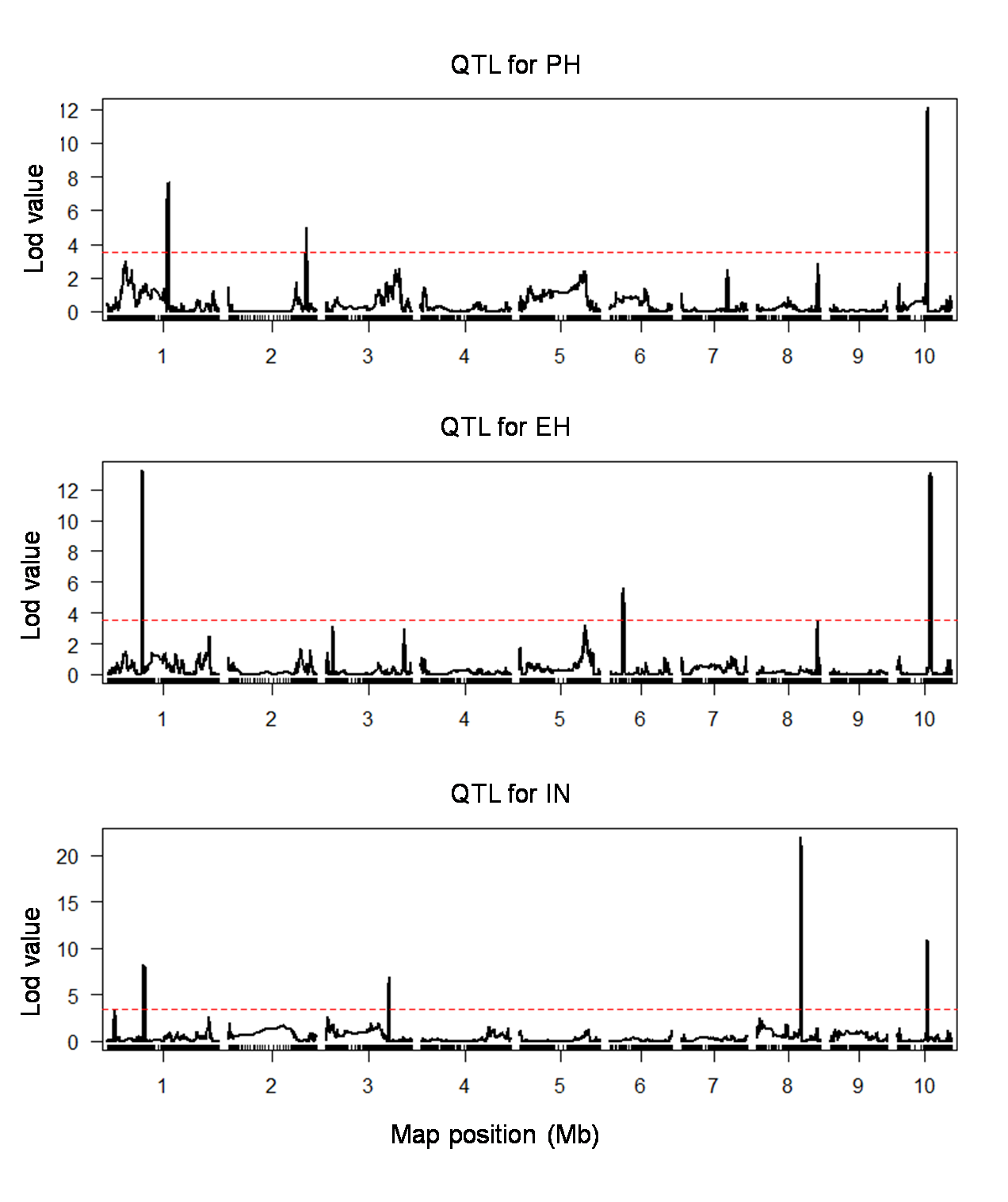


**Figure S8 Mapping of QTL for PH, EH, and IN on the ten maize chromosomes in three environments combined.** The curves indicate the physical position (x-axis) of bin markers against the LOD scores (y-axis) of QTL detected on each of ten chromosomes. The red dashed lines represent the LOD threshold. PH: plant height; EH: ear height; IN: internode number.

**Table S1 Detailed information regarding the 17 bins that were greater than 10 Mb in length**

| **Marker name** | **Chr.^1^** | **Physical position (Mb)** | **Bin length (Mb)** | **Description** | **Centromeric position**  **(B73 RefGen_V3)** |
| --- | --- | --- | --- | --- | --- |
| mk306 | 1 | 135.9 | 10.8 | centromeric | 120,194,433 –143,517,797 |
| mk849 | 2 | 108.75 | 18.5 | pericentromeric | 71,679,747 – 100,074,126 |
| mk855 | 2 | 160.35 | 12.9 |  |
| mk853 | 2 | 147.65 | 11.5 |  |
| mk842 | 2 | 57.25 | 10.3 | pericentromeric |
| mk1228 | 3 | 69.3 | 16.8 | pericentromeric | 95,266,767 – 110,673,280 |
| mk1236 | 3 | 98.1 | 11.2 | centromeric |
| mk1704 | 4 | 115.4 | 12.2 | centromeric | 96,312,190 – 118,324,906 |
| mk2228 | 5 | 102.6 | 17.2 | pericentromeric | 135,854,897 – 147,481,677 |
| mk2229 | 5 | 117.25 | 12.1 | pericentromeric |
| mk2536 | 6 | 51.95 | 12.3 | centromeric | 48,799,200 – 60,173,502 |
| mk2512 | 6 | 23.25 | 10.7 | pericentromeric |
| mk2949 | 7 | 63.45 | 15.3 | pericentromeric | 50,148,384 – 51,649,968 |
| mk3305 | 8 | 61.55 | 13.3 | pericentromeric | 34,624,883 – 39,211,466 |
| mk3295 | 8 | 34.5 | 11.2 | centromeric |
| mk3995 | 10 | 47.3 | 21 | centromeric | 42,409,158 – 62,253,537 |
| mk3996 | 10 | 64.65 | 13.7 | centromeric |

^1^Chr., indicates chromosome.

**Table S2 QTL identified on the ten maize chromosomes for PH, EH, and IN by combined analysis in three environments**

| **Trait Name^1^** | **Name^2^** | **Chr.^3^** | **Flanking** | **Interval^5^** | **Physical Length^6^(Mb)** | **LOD^7^** | **PVE^8^** | **ADD^9^** |
| --- | --- | --- | --- | --- | --- | --- | --- | --- |
|  |  |  | **Marker^4^** | **(Mb)** |  |  |  |  |
| PH | *qPH1* | 1 | mk328–mk335 | 161.60–168.10 | 7.50 | 7.64 | 9.49 | 11.15 |
|  | *qPH2* | 2 | mk966–mk977 | 209.7–212.35 | 2.65 | 5.01 | 5.39 | 8.40 |
|  | *qPH10* | 10 | mk4016–mk4026 | 81.30–85.10 | 3.80 | 12.14 | 12.82 | 12.86 |
| EH | *qEH1* | 1 | mk271–mk280 | 92.05–98.35 | 6.30 | 13.27 | 13.12 | 7.45 |
|  | *qEH6* | 6 | mk2524–mk2534 | 34.10–40.05 | 5.95 | 5.62 | 3.88 | 4.13 |
|  | *qEH10* | 10 | mk4030–mk4037 | 87.05–94.45 | 7.45 | 13.10 | 13.44 | 7.58 |
| IN | *qIN1* | 1 | mk277–mk284 | 97.05–102.05 | 5.00 | 8.26 | 5.26 | 0.43 |
|  | *qIN3* | 3 | mk1347–mk1365 | 169.50–174.65 | 5.15 | 6.92 | 5.90 | -0.45 |
|  | *qIN8* | 8 | mk3399–mk3409 | 119.75–123.4 | 3.65 | 21.96 | 20.13 | -0.83 |
|  | *qIN10* | 10 | mk4016–mk4026 | 81.75–85.05 | 3.30 | 10.93 | 9.70 | 0.58 |

^1^Trait is the name of the component of plant architecture: PH, plant height; EH, ear height; IN, internode number.

^2^The name of each QTL is a composite of the influenced trait: PH, EH, IN.

^3^Chr., chromosome.

^4^Flanking Markers, the markers to the left and right of the QTL.

^5^Interval, confidence interval between two bin markers.

^6^Physical Length, interval between the two markers on the B73 genome.

^7^LOD, the logarithm of odds score.

^8^PVE, the phenotypic variance explained by individual QTL.

^9^ADD, the additive effect value.

**Table S3 Genes located in the intervals of *qPH10***

| **Chr.^1^** | **Pos. start^2^** | **Pos. end^3^** | **Gene ID** | **Annotation** |
| --- | --- | --- | --- | --- |
| 10 | 81700345 | 81730386 | GRMZM2G108892 | myb-like transcription factor family protein |
| 10 | 81839592 | 81844874 | GRMZM2G325907 | (AtMYB103, MYB103) myb domain protein 103 |
| 10 | 81969724 | 81974407 | GRMZM2G070780 | Erythronate-4-phosphate dehydrogenase family protein |
| 10 | 82037179 | 82043124 | GRMZM5G832300 | RING/U-box superfamily protein |
| 10 | 82056684 | 82059708 | GRMZM2G358161 | (NIP5;1, NLM6, NLM8) NOD26-like intrinsic protein 5;1 |
| 10 | 82058765 | 82065097 | GRMZM5G856084 | DNAJ heat shock family protein |
| 10 | 82051086 | 82057877 | GRMZM5G828229 | (MDAR6) monodehydroascorbate reductase 6 |
| 10 | 82112652 | 82116777 | GRMZM2G341304 | (RLK) receptor lectin kinase |
| 10 | 82120517 | 82143616 | GRMZM2G042980 | N/A |
| 10 | 82164257 | 82167174 | AC188036.3_FGT003 | N/A |
| 10 | 82169212 | 82181242 | GRMZM2G032684 | Oxidoreductase family protein |
| 10 | 82217460 | 82223873 | GRMZM2G149786 | (LDL2) LSD1-like2 |
| 10 | 82247382 | 82250336 | GRMZM2G415470 | N/A |
| 10 | 82271020 | 82277588 | GRMZM2G433727 | (RAD23A) Rad23 UV excision repair protein family |
| 10 | 82349699 | 82353841 | GRMZM2G452084 | N/A |
| 10 | 82369728 | 82373770 | GRMZM2G172139 | N/A |
| 10 | 82373088 | 82381551 | GRMZM2G172153 | (ATRPL23A, RPL23A, RPL23AA) ribosomal protein L23AA |
| 10 | 82398998 | 82402602 | AC193645.3_FGT006 | N/A |
| 10 | 82401920 | 82410383 | GRMZM2G178942 | (ATRPL23A, RPL23A, RPL23AA) ribosomal protein L23AA |
| 10 | 82627047 | 82631215 | GRMZM2G054309 | Protein of unknown function (DUF594) |
| 10 | 82681153 | 82684288 | GRMZM2G121137 | N/A |
| 10 | 82696420 | 82700069 | GRMZM2G701134 | SIT4 phosphatase-associated family protein |
| 10 | 82704630 | 82707181 | GRMZM2G362828 | N/A |
| 10 | 82727827 | 82735975 | GRMZM2G063363 | PapD-like superfamily protein |
| 10 | 82849995 | 82854273 | GRMZM2G021388 | Pyridoxal phosphate (PLP)-dependent transferases superfamily protein |
| 10 | 82861182 | 82865312 | GRMZM2G021277 | Pyridoxal phosphate (PLP)-dependent transferases superfamily protein |
| 10 | 82941026 | 82945770 | GRMZM2G339180 | (OVA2) tRNA synthetase class I (I L M and V) family protein |
| 10 | 83096225 | 83099808 | GRMZM2G309126 | (ATTPS21, TPS21) terpene synthase 21 |
| 10 | 83107498 | 83112007 | GRMZM2G016254 | (ATTPS21, TPS21) terpene synthase 21 |
| 10 | 83324101 | 83327881 | GRMZM2G304010 | (B80, PUB8) plant U-box 8 |
| 10 | 83430040 | 83434556 | GRMZM2G003765 | plastid developmental protein DAG putative |
| 10 | 83459738 | 83469025 | GRMZM2G003897 | RNA-binding (RRM/RBD/RNP motifs) family protein |
| 10 | 83468813 | 83471900 | GRMZM2G306105 | N/A |
| 10 | 83550464 | 83558118 | GRMZM2G393742 | (CHR35, DMS1, DRD1) SNF2 domain-containing protein / helicase domain-containing protein |
| 10 | 83560419 | 83566227 | GRMZM2G093858 | (CRLK1) Protein kinase superfamily protein |
| 10 | 83605148 | 83610440 | GRMZM2G093744 | (bHLH105, ILR3) basic helix-loop-helix (bHLH) DNA-binding superfamily protein |
| 10 | 83680541 | 83698115 | GRMZM2G060054 | (AVA-P4) vacuolar H+-pumping ATPase 16 kDa proteolipid subunit 4 |
| 10 | 83752307 | 83757619 | GRMZM2G103785 | Protein of unknown function (DUF604) |
| 10 | 83846901 | 83850001 | GRMZM2G148884 | (UCC1) uclacyanin 1 |
| 10 | 84033426 | 84036471 | GRMZM2G429163 | (AtRLP26, RLP26) receptor like protein 26 |
| 10 | 84042613 | 84046592 | GRMZM2G002818 | (GSO1) Leucine-rich repeat transmembrane protein kinase |
| 10 | 84105702 | 84111776 | GRMZM2G148924 | (FIE, FIE1, FIS3) Transducin/WD40 repeat-like superfamily protein |
| 10 | 84241542 | 84246926 | GRMZM2G004957 | (PDF2) protodermal factor 2 |
| 10 | 84246242 | 84251053 | GRMZM2G005024 | (ATTSB1, TRP2, TRPB, TSB1) tryptophan synthase beta subunit 1 |
| 10 | 84251239 | 84260713 | GRMZM2G005126 | Zinc finger C-x8-C-x5-C-x3-H type family protein |

^1^Chr., indicates chromosome.

^2^Pos. start, the start position of the gene.

^3^Pos. end, the end position
